# Mixed Feedings and Necrotizing Enterocolitis: The Proportion of Human Milk Matters

**DOI:** 10.1089/bfm.2022.0268

**Published:** 2023-06-15

**Authors:** Xiaoyun Xiong, Yanqiong Wang, Xueyu Chen, Bingchun Lin, Yanzhu Zhuang, Li Luo, Huiyan Wang, Chuanzhong Yang

**Affiliations:** ^1^ Department of Neonatology, Affiliated Shenzhen Maternity and Child Healthcare Hospital, The First School of Clinical Medicine, Southern Medical University, Shenzhen, China.; ^2^ School of Nursing, Philippine Women's University, Manila, Philippines.; ^3^ Shanxi University of Chinese Medicine, College of nursing, Jinzhong, China.

**Keywords:** human milk, feeding intolerance, proportion, necrotizing enterocolitis

## Abstract

**Objectives:**

Impact of the proportion of human milk (HM) in mixed feeding on necrotizing enterocolitis (NEC) remains unknown. This study explores the influence of different proportions of HM on the risk of NEC.

**Materials and Methods:**

A retrospective cohort study was performed in infants with very low birth weight (VLBW). A spline smoothing curve was used to evaluate the dose-dependent association between HM and the risk of NEC. Univariate and multivariate analyses were performed to detect the association between the proportion of HM and NEC.

**Results:**

Twenty-four infants developed NEC, with 4 (1.9%) in the high HM group, 18 (28.1%) in the low HM group, and 2 (8.0%) in the exclusive formula group (*p* < 0.001). After adjusting for the relevant confounders, low HM (proportion of HM ≤54%) (OR 33.526, 95% confidential interval [CI] 7.183–156.475, *p* < 0.001) and exclusive formula feeding (OR 8.493, 95% CI 1.107–65.187, *p* = 0.040) significantly increased the incidence of NEC, compared with the high HM feeding (proportion of HM >54%). Similarly, low HM was independently associated with an increased risk of feeding intolerance compared with high HM feeding (OR 4.383, 95% CI 2.243–8.564, *p* < 0.001).

**Conclusion:**

A low ratio of HM (≤54%) significantly increased the risk of intestinal complications in VLBW infants. Mixed feeding should relate to the proportion of HM in premature infants.

## Introduction

Necrotizing enterocolitis (NEC) is a devastating intestinal disease leading to neonatal death in infants born prematurely.^1,2^ Approximately 7% of infants with very low birth weight (VLBW) develop into NEC.^3^ In VLBW infants with NEC, the mortality is up to 20–30%, and long-term sequelae of neurodevelopmental impairment and short bowel syndrome are prevalent in the survivors.^4^ Although the leading cause of NEC remains elusive, human milk (HM) significantly reduces the risk of NEC.^5–7^ Several studies have demonstrated a protective role of exclusively HM feeding.^8,9^

However, it is expected that mothers of premature infants admitted to neonatal intensive care unit (NICU) often cannot provide sufficient HM. The newborns have to receive a certain amount of formula feeding, especially in regions where donor HM is not available.^10^ Whether combining a limited amount of HM with formula also prevents NEC remains controversial. Some researchers reported that any amount of HM would be more beneficial than exclusive formula feeding,^9^ while others claimed that only a high proportion of HM could decrease the risk of NEC.^11^ In this study, we aim to explore the influence of different HM proportions on NEC in a retrospective cohort of VLBW infants.

## Materials and Methods

### Study design

The current study retrospectively analyzed clinical data of VLBW infants admitted to the NICU from July 2018 to June 2019. Infants with congenital genetic metabolic diseases, developmental malformations, early sepsis (confirmed by culture), intestinal perforation within 72 h after birth, and death within 2 weeks after hospitalization were excluded. Trophic feeding was initiated 24 hours after admission and maintained for 1 week, mainly via nasogastric (gavage) feeding. From the second week, the milk intake was routinely increased at a speed of 1–2 mL/kg/meal if the infants could tolerate the feeding. All mothers were encouraged to give their milk to their babies. Donor milk was not available in our NICU. Formula feeding was used if HM was unavailable or not enough. The Ethics Committee approved the study protocol (No. [2019]-034) and waived the informed consent since no identifying information was collected.

### Data collection

Clinical data were collected, including gestational age, birth weight, gender, intrauterine distress, antenatal steroids exposure, method of delivery, Apgar at 1- and 5-minutes, surfactant replacement, duration of intubation, duration of noninvasive ventilation, empirical antibiotics, probiotics, fortifier, and duration of hospital stay. Besides, the intake of HM or formula was recorded daily till 14 days after birth. Postnatal life of 14 days was chosen based on the study of Meinzen-Derr et al., where they reported that HM feeding for 14 days was enough to yield a protective effect on NEC.^12^ The proportion of HM was calculated as the total amount of HM intake divided by the total volume of enteral feeding within 14 days.

### Definition

NEC is the primary outcome of the cohort and is defined as Bell's stage II or III.^1^ Feeding intolerance was diagnosed when infants had one of the following symptoms: increased gastric residual volume (defined as gastric residual >30% of the feeding volume and more than that from the last feeding or >50% of the feeding volume), abdominal distension, vomiting, and bloody stools (except for sepsis with positive blood culture and NEC).^13^ Bronchopulmonary dysplasia (BPD), retinopathy of prematurity (ROP), and intraventricular hemorrhage (IVH) were diagnosed as previously reported.^14–16^

### Statistics

The sample size calculation was based on the prevalence of NEC in our unit. The rate of NEC in exclusive formula-fed infants is around 8%, 2% in high HM-fed (>54%) ones, and 20% in low HM-fed (≤54%) infants. At 80% power and α = 0.05, 271 infants at most would be sufficient to detect a significant difference among the three groups (PASS, Version 11; NCSS, LLC, UT). A two-piecewise linear regression model was performed to examine the threshold effect of the HM proportion on NEC risk using a smoothing function, using Empower(R); X&Y solutions, Inc., Boston, MA) and R Project. SPSS (V24.0; IBM, Armonk, NY) was used for statistical analysis. Continuous data expressed as mean (standard deviation) or median (interquartile range) were analyzed by one-way analysis of variance or nonparametric analysis for comparison among multiple groups.

Categorical data were presented as frequency and percentage and analyzed by *χ*^2^ tests or the Fisher's exact test when appropriate. Multivariate logistic regression analysis was used to detect the independent association between the proportion of HM and NEC or feeding intolerance. The receiver operating characteristic (ROC) curve was further applied to assess the cutoff value of HM proportion in mixed feeding to discriminate VLBW infants developing into NEC. A *p*-value <0.05 was considered statistically significant.

### Ethical statement

The study was performed in accordance with the Declaration of Helsinki and approved by the Ethics Committee (IEC), who waived the requirement for informed consent (no. [2019]-034).

## Result

During the study period, 321 VLBW infants were admitted to the NICU within 24 hours after birth. Eighteen infants were excluded from this study, of whom 3 infants were diagnosed with intestinal perforation within 72 hours, 12 babies died within 72 hours of hospitalization, and 3 died within 2 weeks after birth. Therefore 303 VLBWs were included in the analysis. Twenty-four infants developed NEC (7.9%) at postnatal day 25 (17–37) or a gestational age of 33 (31.4–34) weeks. The clinical characteristics are summarized in Table 1.

**Table 1.** Comparison of Clinical Characteristics of Different Groups

Variable	High HM (>54%, 214)	Low HM (≤54%, 64)	Formula (25)	*F*/*χ*^2^*^a^*	*p^a^*
Male	119 (55.6%)	38 (59.4%)	16 (64.0%)	0.816	0.665
Gestational age (week)	29.2 ± 2.9	29.0 ± 2.7	29.6 ± 2.3	0.620	0.734
Birth weight (g)	1176 ± 233	1148 ± 260	1176 ± 183	0.404	0.817
Vaginal delivery	80 (37.4%)	25 (39.1%)	8 (32.0%)	0.386	0.825
Intrauterine distress	16 (7.5%)	7 (10.9%)	2 (8.0%)	0.782	0.676
Antenatal steroids	108 (50.5%)	42 (65.6%)	16 (64.0%)	6.058	0.048
1-minute Apgar score^b^	9 (7, 10)	9 (7, 10)	9 (8, 10)	11.002	0.894
5-minute Apgar score^b^	10 (10, 10)	10 (10, 10)	10 (10, 10)	8.533	0.742
Surfactant	116 (54.2%)	31 (48.4%)	13 (52.0%)	0.665	0.717
Intraventricular hemorrhage	30 (14.0%)	7 (10.9%)	5 (20.0%)	1.252	0.535
Intubation (days)	2.3 ± 7.4	2.6 ± 7.0	2.9 ± 13.2	0.939	0.625

High HM, the proportion of HM more than 54%. Low HM, the proportion of HM less than 54%.

^a^ comparison among the three groups.

^b^ One missing data in the high HM group.

HM, human milk.

### Relationship between HM proportion and risk of NEC

**FIG. 1.**
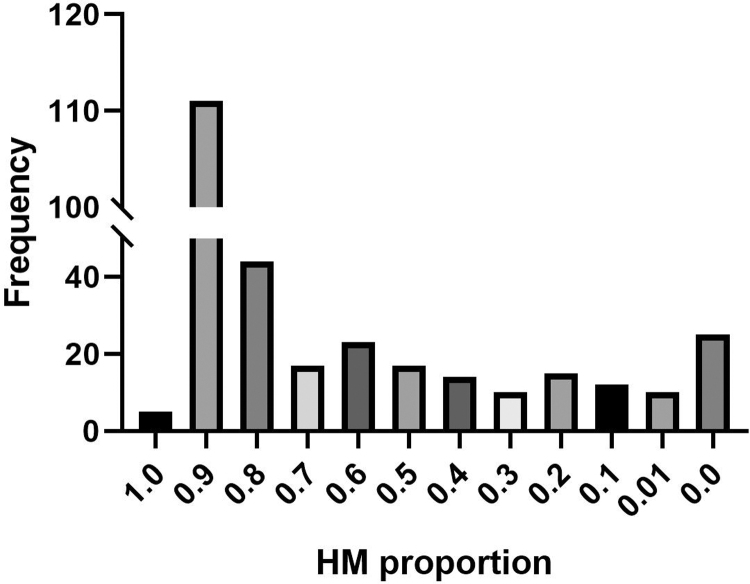
Distribution of infants numbers receiving variated proportions of HM. The ratio in *X*-axis indicates a range between it and the ratio in front. For example, the **column** of 0.9 represents a range between 0.90 and 0.99. Columns 0.0 and 1.0 stands for exclusive formula and human milk, respectively. HM, human milk.

**FIG. 2.**
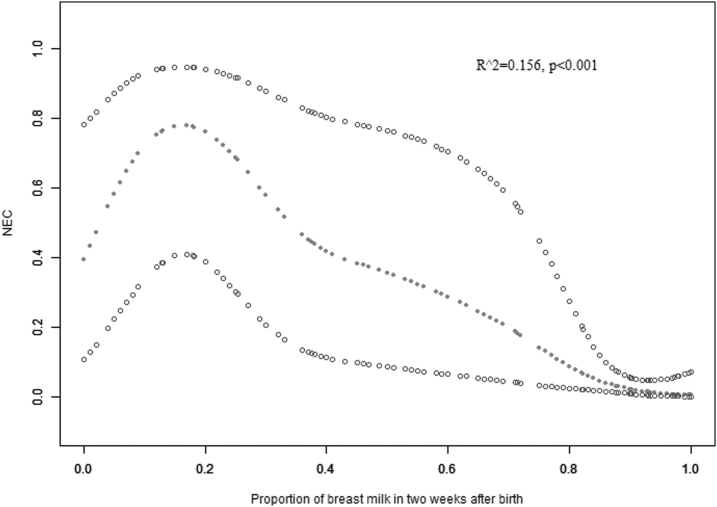
Association of HM proportion with NEC risk. Unadjusted data for the rate of NEC plotted against HM proportion within 2 weeks after birth and fitted with a curve indicating a two-piecewise relationship between HM proportion and NEC risk. Fitting curve (the **middle line**) and the 95% confidential interval (the **upper** and **lower lines**). HM, human milk; NEC, necrotizing enterocolitis.

The distribution of HM proportion within 14 days was demonstrated in Figure 1. A two-piecewise linear regression model was conducted to depict the dose-dependent relationship between HM proportion and risk of NEC using a smoothing function (Fig. 2). Of note, the risk for NEC increased from around 40% to near 80% when HM rose from 0 to ∼20%. With increasing the proportion of HM, the risk of NEC decreased. At around 50%, the risk of NEC decreased to 40%, close to the risk with exclusively formula feeding.

**FIG. 3.**
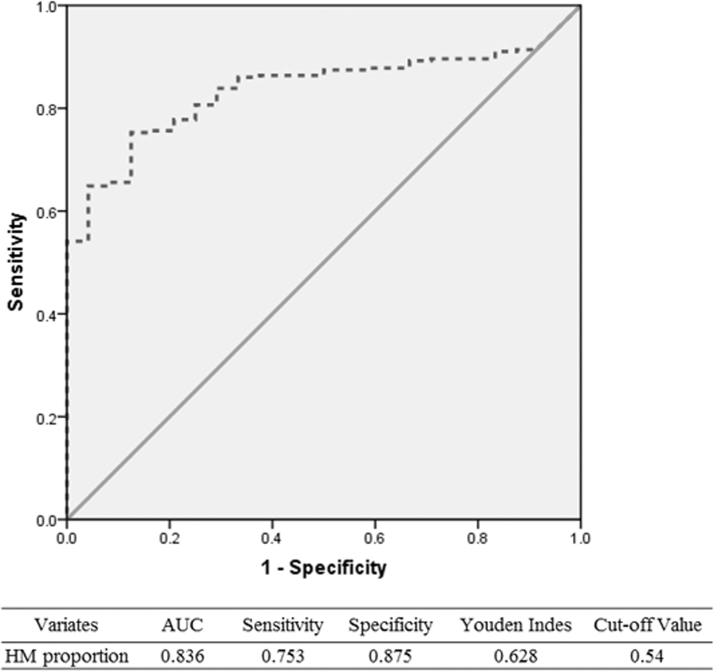
Receiver operating characteristic curve and calculation of the cutoff value for HM proportion. A receiver operator curve was performed and the cutoff value of the HM proportion within the first 2 weeks after birth optimally predicting the necrotizing enterocolitis was calculated. A HM proportion of 54% was concluded as the cutoff value, with an area under the curve of 0.836, a sensitivity of 0.753, a specificity of 0.875, and a Youden index of 0.628. HM, human milk.

Therefore, the cohort was divided into three groups based on the cutoff value calculated from the ROC curve, high HM group (HM proportion >54%, 214 infants), low HM group (HM proportion ≤54%, 64 infants), and exclusive formula group (25 infants; Fig. 3). The rate of NEC was 1.9% (4/214) in the high HM group, 28.1% (18/64) in the low HM group, and 8.0% (2/25) in the exclusive formula group, *p* < 0.001. Similarly, a significant difference was observed in the rate of feeding intolerance in these groups (20.6%, 60.9% and 36.0%, *p* < 0.001). No significant difference was found in the rate of BPD, ROP, and IVH at III or IV stages (Table 2).

**Table 2.** Outcomes of Different Groups

Variable	High HM (214)	Low HM (64)	Formula (25)	*F*/*χ*^2a^	*p^a^*
NEC	4 (1.9%)	18 (28.1%)	2 (8.0%)	46.566	<0.001
Feeding intolerance	44 (20.6%)	39 (60.9%)	9 (36.0%)	38.396	<0.001
BPD	75 (35.0%)	26 (40.6%)	10 (40.0%)	0.793	0.683
ROP	41 (19.2%)	12 (18.7%)	4 (16.0%)	0.146	0.971
IVH III/IV	3 (1.4%)	2 (3.1%)	0 (0%)	1.210	0.566

NEC defined as Bell's stage II or III. High HM, the proportion of HM more than 54%. Low HM, the proportion of HM less than 54%.

^a^ Comparison among the three groups.

BPD, bronchopulmonary dysplasia; HM, human milk; NEC, necrotizing enterocolitis; ROP, retinopathy of prematurity; IVH III/IV, grade III or IV intraventricular hemorrhage.

### The impact of HM proportion on NEC and feeding intolerance

Univariate analysis was performed to evaluate the association between risk factors and NEC or feeding intolerance (Supplementary Table S1). After adjusting for the relevant confounders, low HM (≤54%) (OR 33.526, 95% confidential interval [CI] 7.183–156.475, *p* < 0.001) and exclusive formula feeding (OR 8.493, 95% CI 1.107–65.187, *p* = 0.040) in the first 2 weeks were significantly associated with a higher incidence of NEC, compared with the high HM feeding (>54%). Similarly, low HM was independently associated with an increased risk of feeding intolerance compared with high HM feeding (OR 4.383, 95% CI 2.243–8.564, *p* < 0.001) (Table 3).

**Table 3.** Multifactor Logistics Regression Analysis of Influencing Factors of Necrotizing Enterocolitis and Feeding Intolerance

Outcome	Groups	Ratio	Predicted probabilities (95%CI)	OR (95% CI)	*p*
NEC	High HM	4/214 (1.9%)	1.0 − 1.2%		
	Low HM	18/64 (28.1%)	22.7 − 29.1%	33.526 (7.183–156.475)	<0.001
	Formula	2/25 (8.0%)	7.3 − 11.8%	8.493 (1.107–65.187)	0.040
Feeding intolerance	High HM	44/214 20.6%)	17.7 − 19.6%		
	Low HM	39/64 (60.9%)	51.7 − 57.3%	4.383 (2.243–8.564)	<0.001
	Formula	9/25 (36%)	27.4 − 29.9%	1.021 (0.347–3.006)	0.970

Multivariate logistic analysis was adjusted with gestational age, birth weight, 1-minute Apgar score, probiotics, fortifier and prolonged empirical antibiotics exposure (longer than 5 days). High HM, the proportion of HM more than 54%. Low HM, the proportion of HM less than 54%. The high HM group was the reference group.

CI, confidence interval; HM, human milk; NEC, necrotizing enterocolitis.

## Discussion

Despite the exclusive and high proportion of HM being proven to reduce the risk of NEC, many mothers may be unable to provide enough HM in the first days after delivery. Therefore, mixed feeding is common in preterm infants where donor milk is unavailable. Whether mixed feeding with a low ratio HM is beneficial or harmful? Is there a threshold? To our knowledge, this study is the first to address these practical questions. The current study found that the relationship between HM proportion within 2 weeks and NEC risk was with a two-piece pattern. To our surprise, the risk of NEC is even higher in a low proportion of HM (≤54%) compared to exclusive formula feeding.

Sisk et al. reported that a higher intake of HM was more effective than a lower intake of HM for preventing NEC.^11^ Similar to this and other studies,^5,12,17,18^ we show that HM intake within 2 weeks after birth negatively correlated with the rate of NEC overall. Low HM and exclusive formula feeding in the first 2 weeks increased the risk for NEC, compared with high HM feeding. Besides, although many previous studies reported longer HM feeding, more HM intake, and earlier exclusive HM feeding confer a better protective effect than shorter, lower, or no HM feeding,^19^ few had compared the difference between low HM intake and exclusive formula feeding. Here, we found a tendency toward increased risk of NEC in infants receiving less than 54% HM of total enteral feeding volume, compared to exclusive formula feeding (>54%, OR 3.947, *p* = 0.098, Supplementary Table S2).

Although meta-analysis concluded that formula feeding increased the risk of NEC compared to either own mother's milk or donor milk,^20,21^ the detailed proportion of HM milk was not reported in those analyses. Some studies compared formula and donor milk;^22^ infants likely have enough access to donor milk in NICUs with donor milk banks. Miller et al. reported that any volume of HM was better than exclusive formula feeding in their meta-analysis.^9^ This finding was concluded from observational studies since no randomized trials were found on this topic. Furthermore, any HM includes both lower HM and high HM. Therefore, the harmful effect of low HM feeding might be outweighed by the beneficial effect of high HM feeding.

Similarly, some studies concluded that higher the dose the greater the protection,^5,22^ while the lower dose was ambiguously defined since the exact amount was either not reported or varied significantly.^9^ Some researchers have suggested that more than 50% of enteral feeding was needed to ensure the protective effect of HM,^5^ while the threshold was casually estimated rather than precisely calculated.^19,23^ In the current study, we identified a cutoff of the proportion of HM and visualized the detrimental effect of low HM on intestinal complications.

HM contains immunomodulating ingredients that help with the maturation of the infant's immune system, such as secretory immunoglobulin A, oligosaccharides, and various growth factors that might reduce the inflammation response and enhance the host defense in the premature infants.^24^ Moreover, HM might reduce NEC via modulation of the gut microbiota.^25–27^ However, why low HM feeding tends to increase the risk of NEC is unknown. A low HM indicates a mixed feeding of HM and formula. A frequent shift between HM and formula might disturb the premature intestine, leading to NEC. Our findings regarding feeding intolerance could prove this. We found that low HM significantly increased the frequency of feeding intolerance by 4.3-fold compared to exclusive formula feeding. Besides, an immunological mechanism could also be involved, while further investigation is needed to explore the underlying mechanism.

Our finding is of particular clinical significance, especially in low-resource settings where preterm infants lack sufficient own mother's milk and might have limited access to human donor milk. Neonatologists must pay extra attention to intestinal symptoms in infants receiving a low proportion of HM (<54%). However, prohibition on low HM feeding in preterm infants is not recommended since a low proportion of HM might benefit preterm infants in other aspects, such as BPD, late-onset sepsis, neurodevelopment, and growth.^28^

The main limitation of our study, apart from the retrospective nature, is the relatively small sample size. We only observed a tendency toward a higher rate of NEC in the low HM group compared to exclusive formula feeding. Besides, this study did not investigate the underlying mechanism of why low HM increased the risk for NEC.

In summary, we have reported an interesting finding that a low proportion of HM (less than 54% of enteral feeding) within the first 2 weeks increased the risk of NEC compared to exclusive formula feeding. Our study suggests that mixed feeding should consider the proportion of HM in premature infants.

## Supplementary Material

Supplemental data

Supplemental data
