# Supplementary material for: Mixed Feedings and Necrotizing Enterocolitis: The Proportion of Human Milk Matters
Source: Breastfeed Med. 2023 Jun 15;18(6):469–74. doi: 10.1089/bfm.2022.0268 (PMC10282811; doi:10.1089/bfm.2022.0268)
Supplement: Supplemental data [file Suppl_TableS1.docx]

Supplemental Table 1

Risk factors of NEC among different groups

| variable | High HM（214） | Low HM（64） | Formula  （25） | F/*χ*^2^*^#^* | *P^#^* |
| --- | --- | --- | --- | --- | --- |
| Fortifier | 146 (68.2%) | 27 (42.2%) | 0 (0%) | 59.816 | <0.001 |
| Probiotics | 88 (41.1%) | 35 (54.7%) | 14 (56.0%) | 6.821 | 0.033 |
| Ibuprofen exposure | 62 (29.0%) | 17 (26.6%) | 4 (16.0%) | 1.922 | 0.383 |
| Prolonged empirical antibiotics exposure | 136 (63.6%) | 38 (59.4%) | 13 (52.0%) | 1.452 | 0.484 |

High HM, the proportion of HM more than 54%. Low HM, the proportion of HM less than 54%. Prolonged empirical antibiotics exposure, defined as empirical antibiotics exposure longer than 5 days. # comparison among the three groups.
