# Supplementary material for: Mixed Feedings and Necrotizing Enterocolitis: The Proportion of Human Milk Matters
Source: Breastfeed Med. 2023 Jun 15;18(6):469–74. doi: 10.1089/bfm.2022.0268 (PMC10282811; doi:10.1089/bfm.2022.0268)
Supplement: Supplemental data [file Suppl_TableS2.docx]

Supplemental Table 2

Multifactor logistics regression analysis of influencing factors of NEC and Feeding intolerance

| Outcome |  | Ratio | *β/OR* (95%CI) | *P* |
| --- | --- | --- | --- | --- |
| NEC | Formula | 2/25 (8.0%) |  |  |
|  | High HM | 4/214 (1.9%) | 0.118 (0.015, 0.904) | 0.040 |
|  | Low HM | 18/64 (28.1%) | 3.947 (0.777, 20.046) | 0.098 |
| Feeding | Formula | 9/25 (36%) |  |  |
| intolerance | High HM | 44/214 (20.6%) | 1.005 (0.340, 2.974) | 0.992 |
|  | Low HM | 39/64 (60.9%) | 4.413 (1.420, 13.722) | 0.010 |

Multivariate logistic analysis was adjusted with gestational age, birth weight, 1-min Apgar score, probiotics, fortifier and prolonged empirical antibiotics exposure (longer than five days). High HM, the proportion of HM more than 54%. Low HM, the proportion of HM less than 54%. The exclusive formula group was the reference group.
